# A Cost-Effective and Rapidly Manufacturable Infrared–Visible High-Contrast Calibration Board Based on Structural Parametrization

**DOI:** 10.3390/jimaging12050199

**Published:** 2026-05-02

**Authors:** Yuandong Shao, Aleksandr S. Vasilev

**Affiliations:** Institute “Higher School of Engineering and Technology”, ITMO University, Saint Petersburg 197101, Russia

**Keywords:** infrared–visible registration, homography, checkerboard calibration, cost-effective calibration, image fusion

## Abstract

The infrared (IR)—visible light (VIS) dual-camera system provides complementary cues for image fusion, but issues such as geometric mismatch caused by different imaging methods, inconsistent resolution/field-of-view, and installation offsets often lead to ghosting and artifacts. This study aims to develop a fast-deployable and repeatable calibration workflow based on cost-effective calibration board. We designed an infrared-visible high-contrast checkerboard plate that can be generated through structural parameterization and efficiently manufactured using Python/OpenSCAD. We also established a corner-based registration pipeline that estimates global homography to align the visible-light images onto the infrared pixel grid for fusion and quantitative evaluation. Experiments conducted in a controlled indoor environment demonstrated stable sub-pixel performance within a range of 1.5–2.5 m, with an average re-projection error of 0.47–0.50 pixels per frame and a 95th percentile lower than 0.51 pixels. The corner position re-projection error test further confirmed stability near image boundaries, with a median value of 0.53–0.63 pixels and a 95th percentile of 0.54–0.64 pixels. Overall, the proposed target design and workflow can achieve practical infrared-visible calibration under typical deployment constraints and have repeatable accuracy, providing geometrically consistent input for subsequent fusion and dataset construction.

## 1. Introduction

**Figure 1:**
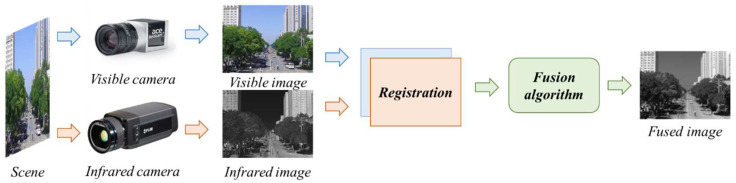
Schematic Diagram of the Infrared and Visible Image Fusion Process. The red and blue arrows represent the acquisition processes for infrared and visible light images, respectively. The green arrow represents the image preprocessing and fusion process.

Infrared (IR) and visible (VIS) imaging, as two complementary visual perception modalities, have become vital data sources for tasks such as security surveillance, unmanned systems, target detection, and scene understanding [1,2,3,4,5,6,7,8]. Visible images typically exhibit richer texture and structural details, while infrared images remain insensitive to illumination variations, providing stable thermal radiation information under challenging conditions such as low light, glare, and smoke. Fusing these two modalities (IR–VIS fusion) simultaneously preserves structural details and thermal target saliency, yielding a more robust and information-dense representation of the scene. Figure 1 presents the typical workflow from acquisition to fusion for infrared and visible images.

However, high-quality fusion in engineering implementation heavily relies on one prerequisite: infrared and visible images must be geometrically registered. In practical systems, infrared and visible light are often captured by two independent optical channels [9]. Due to constraints such as lens focal length, field of view (FOV), sensor resolution, imaging chain processing (denoising/gain/gamma/interpolation), and mechanical assembly errors, residual registration errors between the two modalities commonly persist even when rigid mounts are used. This registration errors may present as global geometric discrepancies (scale shifts, rotations, translations, projection distortions, etc.) or manifest locally as disparities related to depth of field, lens distortion, and parallax caused by differing viewpoints. Without effective geometric compensation, fusion results often exhibit artifacts such as ghosting, edge misregistration, structural shifting, and mismatched texture-thermal information, significantly degrading fusion quality and compromising the reliability of subsequent visual tasks.

Under the current dataset-driven paradigm of deep learning-based fusion, geometric mismatch not only degrades the visual quality of individual fused images but also corrupts the training signal. Many learning-based fusion methods implicitly assume that the IR–VIS training pairs are precisely registered. In practice, however, residual misregistration is common, arising from acquisition imperfections and inconsistent preprocessing such as cropping, scaling, and resampling. When unaligned pairs are used for end-to-end training, the fusion network is forced to absorb misregistration as part of the learning objective: pixel-wise correspondences become unreliable, and structural supervision based on high-frequency cues (e.g., gradients, edges, or Laplacian details) is optimized against spatially shifted targets. This results in supervision noise at the pixel level, which weakens edge-consistency constraints and encourages the network to allocate capacity to reducing apparent misregistration artifacts instead of learning modality-complementary feature integration. Therefore, geometric consistency should be treated as a prerequisite for both fusion quality and reliable learning, rather than a secondary disturbance.

For IR–VIS geometric registration, classical automatic registration typically employs a “feature detection–matching–geometric estimation” pipeline (e.g., cross-modal registration based on SIFT [10,11]). However, cross-modal matching is inherently challenging: differing contrast mechanisms between infrared and visible light, varying texture visibility, and potentially non-overlapping salient regions lead to reduced feature repeatability, increased mismatches, and potential domination of geometric estimation by extreme outliers. This can result in failure or local misregistration.

Reliable geometric calibration between visible and thermal cameras is critically dependent on calibration targets that remain detectable across spectral bands. Prior studies have shown that calibration performance is strongly influenced by target contrast-to-noise ratio in the thermal band and by the robustness of the feature detector, where contrast enhancement and carefully chosen target patterns can significantly improve detection stability under LWIR imaging [12]. In addition, several target designs introduce dedicated thermal structures (e.g., multilayer layouts or deliberate separation/gaps) to mitigate heat cross-talk between adjacent elements, thereby preserving sharp boundaries and improving subpixel localization of features. While these designs can deliver very low reprojection errors under controlled conditions, they often require specialized materials, active thermal control, or more elaborate fabrication [13]. Motivated by practical deployment needs, our work instead focuses on a low-cost, rapidly manufacturable calibration board that can be generated via parametric modeling (Python/OpenSCAD) and produced with minimal tooling, aiming at stable and reproducible geometric registration for downstream IR–VIS fusion and dataset construction. The integrated development environment used for the Python code is PyCharm Community Edition 2024.1.4, with Python version 3.8.20. OpenSCAD (https://openscad.org) was developed and edited directly on the MakerWorld parametric model editing website, as mentioned in the Data Availability Statement.

Existing multispectral calibration studies demonstrate that reliable IR–VIS registration hinges on the cross-spectral observability of calibration targets and the stability of feature localization. However, infrared targets are often constrained by radiometric contrast formation, thermal diffusion at material boundaries, and the practical complexity of heating or active projection setups. Therefore, instead of competing for the lowest possible reprojection error under specialized laboratory hardware, this study emphasizes a rapid and reproducible target-generation workflow for engineering deployment. We propose a simple IR–VIS checkerboard design optimized for multi-material fabrication and thermal/visual observability, and we provide an end-to-end calibration pipeline that can be reproduced with common tools and low-cost manufacturing.

## 2. Related Work

### 2.1. Target-Based Calibration in the Visible Spectrum

Planar calibration targets remain the mainstream choice for visible cameras due to their simplicity, low cost, and high detection accuracy [14,15,16,17]. Classic planar patterns such as chessboards and circle grids provide dense feature correspondences and support well-established intrinsic/extrinsic estimation pipelines (e.g., Zhang-style calibration [18]). In practice, calibration accuracy is heavily influenced by feature localization quality, image noise, and target manufacturability, and these factors become more critical when targets are reused across multiple sessions and working distances. Therefore, this experiment builds upon the design of a stable planar calibration board, ensuring that it remains detectable under both infrared and visible-light imaging while maintaining stability, and facilitating the customization of manufacturing parameters.

### 2.2. Infrared/LWIR Calibration Targets

In contrast to VIS/NIR cameras, LWIR cameras perceive radiance rather than color reflectance; thus, calibration targets must be designed to yield radiometric contrast. Schramm et al. emphasized that while printed chessboards are suitable for VIS calibration, infrared targets exhibit broad diversity and their calibration quality depends strongly on the contrast-to-noise ratio (CNR) and the choice of feature detection algorithm; they further highlight the need to analyze target design and detectability in LWIR/MWIR settings [19].

A common research direction is to generate thermal contrast using emissivity differences, external heating, or layered structures. However, prior studies report that heat diffusion and thermal interference at adjacent cells can blur boundaries, reduce local gradients, and degrade corner/feature localization, especially under non-uniform heating [13]. To address such issues, Sun et al. proposed a dedicated LWIR–VIS calibration plate combining a heating plate and a hollow circular pattern, and they used contour-based center estimation with least-squares fitting (circle/ellipse) to improve robustness under LWIR imaging (the reprojection error is approximately 0.2 pixels).

These works indicate that infrared target design is not merely a “pattern printing” problem; it is coupled with material emissivity, thermal conductivity, heating uniformity, and environmental radiation, all of which jointly determine feature detectability and calibration stability. At the same time, the calibration board approach described above primarily relies on CNC machining of metal materials and specialized processes, which takes several days to complete. Furthermore, it cannot be rapidly deployed when custom dimensional parameters are required. In contrast, existing 3D printers can produce calibration boards in a matter of hours. Combined with our parametric improvements to the calibration board structure, this enables targeted optimizations for camera modules with different focal lengths, resolutions, and fields of view.

### 2.3. Summary

Prior multispectral calibration studies demonstrate that high precision can be achieved when targets provide strong cross-spectral observability and feature extraction is stable. Nevertheless, many solutions rely on specialized hardware (heating plates, controlled thermal separation, active patterns) or fabrication steps that may be costly or difficult to reproduce in everyday engineering workflows. At the same time, Schramm et al. explicitly point out that target detectability and algorithm choice are dominant factors behind calibration quality variations across spectral bands [13].

Compared to cross-spectral calibration targets that rely on specialized heating plates, active projection, or precision machining, the multi-material planar checkerboard calibration plate proposed in this study places greater emphasis on engineering reproducibility and cost-effective deployment. The calibration plate geometry is generated directly from a parametric model, allowing for rapid adjustment and output of the model based on the printer’s build volume and the target plate dimensions. The manufacturing process relies solely on common desktop 3D printers and standard materials (such as PETG), eliminating the need for additional metal machining, embedded heat sources, or synchronized projection systems, thereby significantly lowering the barriers to materials and equipment. During on-site deployment, the system requires only standard incandescent or halogen lamps to provide stable radiant heating, the creating temperature contrast that can be observed in the LWIR channel while maintaining high-contrast boundaries in the VIS channel. This compresses the calibration workflow “target fabrication, placement, thermal equilibrium, data acquisition, corner detection, and monometric estimation” into a process that can be completed in minutes. Compared to highly complex solutions, the primary advantages of this method lie in its more controllable manufacturing costs and time expenditures, reduced reliance on external equipment, and the ease of rapidly replicating and replacing targets. The trade-off is greater sensitivity to close-range field-of-view cropping, heating uniformity, and assembly flatness; however, provided the target is fully within the field of view and thermal contrast is stable, sub-pixel-level alignment accuracy suitable for fusion registration can still be achieved.

## 3. Camera Model

The detailed procedures of the two registration methodologies will be elaborated on in Section 3, while the specifications of the camera module employed in this study are comprehensively documented in Table 1. The LWIR images were acquired using an A615 thermal camera (FLIR Systems, Inc., Wilsonville, OR, USA).The visible images were captured using an acA1300-30gc camera (Basler AG, Ahrensburg, Germany).

**Figure 2:**
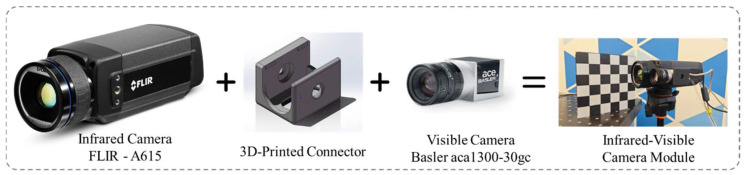
Infrared and visible multispectral camera module prototype.

Figure 2 simultaneously illustrates the prototype module integrating the infrared and visible-light cameras, along with the structural housing that secures the two sensors. Ribs and perforations were distributed across the geometry to enlarge the convective surface area while maintaining adequate mechanical stiffness and mounting stability, thereby ensuring thermal regulation and the stable operation of the visible camera.

In the infrared and visible camera modules shown in Figure 2, it can be observed that the sensor positions of the infrared camera and the visible-light camera differ significantly. Additionally, our study employs a typical non-uniform IR–VIS dual-camera configuration for experimentation. Consequently, infrared and visible cameras typically exhibit technical specifications inconsistencies—such as pixel size, resolution, and lens focal length/field of view—due to differing imaging mechanisms. This paper completes calibration and registration evaluation without enforcing uniform hardware specifications, thereby approximating real-world engineering deployments and validating the method’s robustness.

## 4. Calibration Methods

Under controlled laboratory conditions, we designed and fabricated a bimodal calibration target that exhibits high contrast in both IR and VIS imaging. The calibration board pattern is designed based on the classic Zhang calibration board [18]. We have designed SCAD and Python code capable of automatically generating this calibration board. This enables rapid output of the calibration board model based on printer dimensions and actual calibration board size. The SCAD files can be directly operated for visualization on the MakerWorld website. The calibration board generation file, infrared and visible acquisition and calibration codes, as well as the SCAD visualization website of MakerWorld are all provided in the data availability statement.

**Figure 3:**
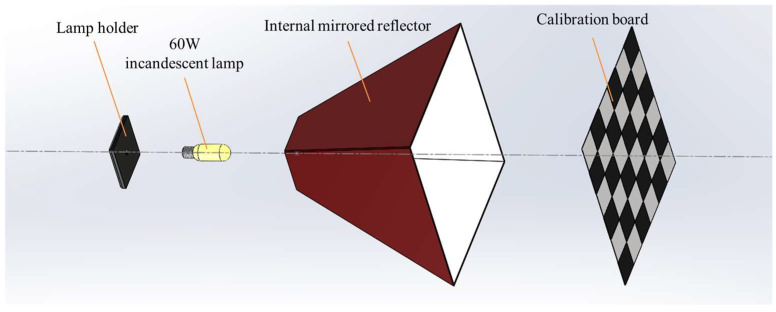
Design of the calibration setup.

The target comprises a 6 × 6 checkerboard pattern produced by 3D printing [20]; detailed specifications are listed in Table 2 and the layered structure is shown in Figure 3. The incandescent lamp used in the experiment operates at a working temperature of approximately 230 °C with 60 W power. The calibration board was fabricated using a multi-material FDM 3D printer (A1, Bambu Lab, Shenzhen, China) and PETG filament (SUNLU (Guangdong) Technology Co., Ltd., Zhongshan, China) and thermally excited using a 60 W incandescent lamp (Navigator Group, Moscow, Russia). Its radiation spectrum covers a continuous wide range from the visible to the infrared wavelength band. Enabling brim printing during the process prevents PETG material warping. Using straight-line infill with 100% density for internal patterns effectively increases heat absorption speed, removes texture from black squares, and reduces error interference during corner point recognition. When illuminated by a wide spectral incandescent lamp, the black and white squares absorb heat at different rates, generating pronounced thermal contrast in the IR image while preserving strong visual contrast in the VIS image. This property enables stable detection in both modalities and supplies an accurate geometric reference. Extracting the checkerboard corners and establishing spatial correspondences yields sub-pixel registration accuracy, making the target well-suited for high-precision indoor calibration tasks in laboratory or industrial environments. Furthermore, when printing the calibration plate, in order to avoid mirror reflection, we did not print on the smooth construction board but instead used a textured PEI construction surface (or an equivalent rough contact surface). Such resulted in a micro-rough and matte surface effect on the printed surface. This surface form can promote diffuse reflection and suppress mirror reflection, thereby reducing sensitivity to environmental heat sources.

Due to the formation of material interfaces at grid boundaries between multiple materials (black/white), interlayer fusion and bonding strength typically prove weaker than in continuous single-material infill regions. Under repeated handling, clamping, or minor bending loads, the stress concentration at these interfaces becomes more prone to cracking. To enhance structural integrity at the boundary, this study introduces an ultra-thin interlayer structure within the black squares: white material replaces black material within a specified height range, creating a “black–white–black” sandwich stacking within the black squares. This design enhances interfacial shear/peel strength by ensuring material continuity across interfaces and facilitating interlayer remelting. Simultaneously, it improves crack and peel resistance at multicolor junctions without significantly increasing overall thickness or mass, thereby boosting the calibration plate’s durability and reusability during experiments.

It is crucial to emphasize that the interlayer material should be white rather than black. Black polymers exhibit stronger absorption and heat accumulation under incandescent lighting; they are prone to localized temperature rises and enhanced thermal diffusion at boundaries. This causes reduced boundary contrast, widened thermal edges, or thermal blooming in infrared imaging, ultimately blurring thermal textures near corners and degrading cross-modal corner/feature detection accuracy.

Regarding parameter settings, the starting height of the intermediate layer should not be set to less than 0.6 mm. If the interlayer appears prematurely, the white material may cause a color-through effect at the bottom of the black squares. This weakens the black–white contrast in the visible light channel and introduces boundary artifact textures, thereby compromising the stability of visible-light corner detection. Conversely, the interlayer should not be more than 0.8 mm: excessive thickness alters the equivalent thermal resistance and heat capacity distribution within the black region, diminishing the black material’s effective heat absorption and thermal transfer to the board surface. This consequently reduces contrast in the infrared channel or distorts the edge thermal gradient morphology. Balancing mechanical strength and imaging contrast, this study adopts an interlayer design characterized by “thin layers, white materials, and restricted height ranges” to achieve equilibrium between structural reliability and cross-modal observability. Regarding the impact of material thickness on calibration effectiveness, we analyzed this from the perspectives of visible light and infrared imaging mechanisms.

**Figure 4:**
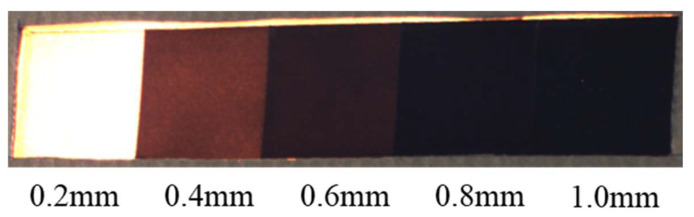
Black material thickness testing when the incandescent lamp is turned on.

In the visible-light channel, checkerboard contrast is primarily determined by the reflective differences between black and white materials. Thickness plays a crucial role in suppressing grayscale elevation caused by light transmission, preventing black squares from appearing gray and white squares from overexposure. This preserves local gradients at corners and maintains detection stability. As shown in Figure 4, we compared material samples of five different thicknesses (0.2–1.0 mm, 0.2 mm increments). The results indicate the following: When black material thickness is insufficient (<0.8 mm), light transmission and brightness increase occur, significantly reducing the grayscale difference between black and white squares and causing insufficient corner contrast. When black material thickness ≥ 0.8 mm, visible-light contrast stabilizes, yielding more reliable corner responses. White material, due to its high reflectivity and pronounced overlap with incandescent light spectrum components, exhibits a relatively weaker thickness-dependent effects on “black–white contrast.” The primary constraint is avoiding excessive thinness that causes light transmission or overexposure. Based on visible-light imaging performance, this study selects a white square thickness of 0.8 mm to ensure adequate brightness while preventing contrast loss from overexposure or light transmission.

**Figure 5:**
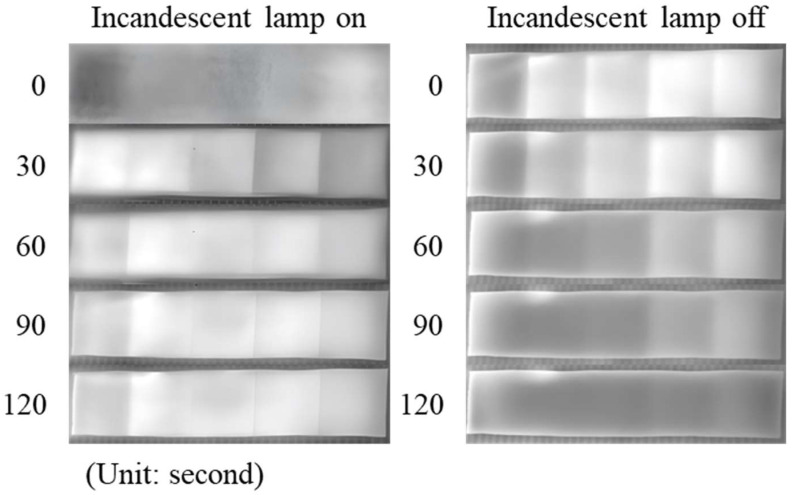
Effect of the thickness of black material on the imaging brightness of infrared cameras; The thickness increases sequentially from left to right (0.2–1.0 mm); Each photo is taken at an interval of 30 s. Numbers 1–5 show the temperature rise after turning on the lamp, while numbers 6–10 show the temperature drop after turning off the lamp.

In the infrared channel, checkerboard contrast does not originate from visible reflectance differences but primarily relies on temperature gradients and thermal radiation variations caused by the heat absorption and temperature rise of black materials under incandescent illumination. Thus, the role of thickness shifts from “light blocking” to a combined effect of “thermal mass and heat conduction pathways.” While thinner samples heat up faster, their heat storage capacity at thermal equilibrium is weaker, and they cool down more rapidly after the light is turned off, leading to unstable infrared contrast. Thicker samples heat up relatively slowly but more easily accumulate and maintain heat, yielding more consistent gray-scale differences under stable or short-term fluctuating illumination. As shown in Figure 5, observations were conducted across two dimensions: time and thickness. The 5 infrared images on the left correspond to the heating process after incandescent lamp activation (recorded every 30 s), where black material temperature rises over time and approaches steady state after approximately 2 min. Near steady state, the 0.2 mm sample heated fastest but exhibited poorer final temperature retention and contrast stability, whereas medium and thicker samples demonstrated more consistent gray-scale differences. The 5 infrared images on the right correspond to the cooling process after lamp shutdown, with thicker samples cooling more slowly. This indicates their superior ability to maintain infrared contrast, thereby enhancing corner point repeatability over time.

Based on the combined constraints of visible light and infrared, this study ultimately determines that the white grid thickness should be 0.8 mm to prevent visible-light overexposure and ensure opacity, while simultaneously defining the permissible thickness range for the interlayer. Considering structural integrity at multicolor print junctions and visible-light appearance consistency, a 0.2 mm thickness is selected for the white interlayer, with its height range set between 0.6 and 0.8 mm. For the black squares, to simultaneously satisfy visible-light shading (preventing contrast reduction) and infrared thermal stability (enhancing heat absorption and insulation), the black material thickness must be ≥0.8 mm. Further balancing thermal capacity and structural integrity, this study sets the total thickness of black squares at 2.0 mm. Approximately 1 mm of solid black material is retained above the interlayer to achieve a compromise between heat absorption and insulation performance. This design maintains relatively stable infrared contrast and corner detectability under varying illumination durations and environmental disturbances.

**Figure 6:**
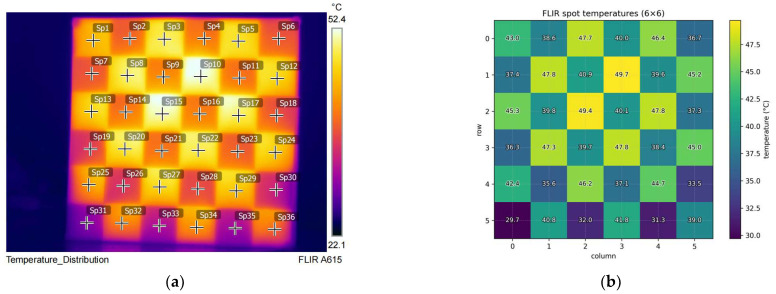
FLIR thermal camera images and temperature distribution simulation. (**a**) FLIR thermal camera test image; (**b**) algorithm-simulated temperature distribution.

To evaluate the effectiveness of the dual-modality chessboard calibration board designed in this paper under LWIR imaging, we used FLIR A615 to sample the temperature field across the calibration board’s surface. Thirty-six sampling points (6 × 6) were selected within the chessboard area according to a regular grid to record temperature readings. Subsequently, a two-dimensional temperature distribution map was constructed to assess thermal contrast and uniformity, as shown in Figure 6. The core objective of this experiment was to confirm whether the calibration plate could generate stable, repeatable thermal contrast boundaries with clear spatial distribution under the infrared camera’s radiation measurement and false-color display mechanism, thereby supporting reliable corner detection and geometric constraint establishment.

**Figure 7:**
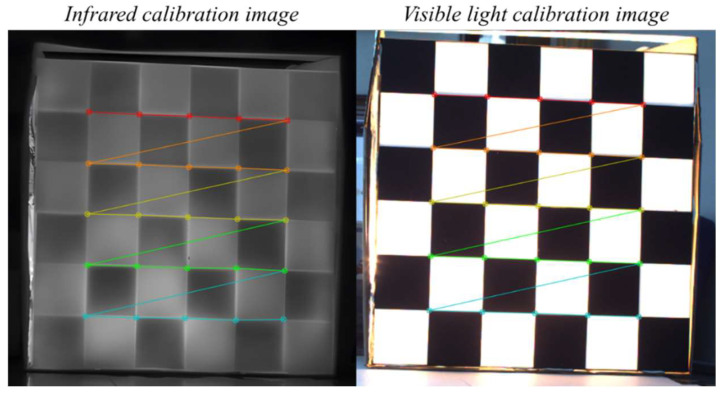
Visualization of calibration board appearance and corner detection in IR and VIS images.

The results demonstrate that the calibration plate exhibits temperature distribution patterns consistent with its checkerboard geometry in the LWIR spectrum: high-/low-temperature regions maintain regular spatial partitioning, with adjacent squares exhibiting distinct thermal radiation differences. After calculating the average values for each black and white square on the chessboard, the average temperature difference was 8.52 °C. This creates clear intensity transitions at grid boundaries within infrared images. Since checkerboard corners correspond to the intersection points of two sets of orthogonal boundaries, these thermal contrast boundaries provide sufficient gradient response and local structural consistency in infrared images. This significantly enhances the detectability and stability of corner localization. In other words, this temperature field distribution validates that the calibration plate is not merely visible in the infrared modality but can form usable features that strictly align with the geometric pattern, satisfying the fundamental requirement of structural observability for calibration/registration algorithms. Preliminary verification of the corner detection experiment results is shown in Figure 7.

While the calibration-board pipeline yields a strongly constrained and highly repeatable solution in controlled, close-range settings, it assumes that a planar target can be placed in the field of view and that sufficient corners are visible without occlusion or cropping.

In summary, the calibration process for converting all subsequent visible-light images to infrared images follows the steps below: First, each infrared and visible image is converted to a single-channel intensity representation and processed with modality-specific contrast enhancement. For LWIR frames, mild denoising and local contrast enhancement are applied to improve boundary gradients under non-uniform heating. For VIS frames, standard grayscale conversion and light smoothing are used to suppress sensor noise. Second, inner chessboard corners are detected using a robust chessboard corner detector with sub-pixel localization capability, and the detected corners are refined using a sub-pixel optimization routine based on local intensity gradients to achieve sub-pixel accuracy. Third, for each image pair, correspondences are established by the known grid ordering of the chessboard, and pairs are accepted only when the detector returns a complete set of valid inner corners (i.e., all m × n corners are detected) and the corner geometry passes basic consistency checks. Fourth, a robust homography estimation strategy is employed using random sample consensus (RANSAC) to reject outliers and prevent occasional corner mis-localization from biasing the global mapping. The estimation uses a defined reprojection threshold and a minimum inlier requirement; frames failing these criteria are discarded. Finally, the estimated global homography is evaluated by computing corner-wise reprojection errors, and we report both per-frame statistics and aggregated metrics (median, interquartile range, and 95th percentile) to characterize accuracy and stability.

## 5. Experiments and Results

In this section, this paper will elaborate on the calibration mechanism of the position around the infrared camera and the visible camera and explore the influence of different distances and different imaging corners on the stability of the calibration results. All experiments were conducted on planar targets with moderate depth variations. Since our downstream goal is to achieve the rapid engineering registration required for fusion rather than complete 3D reconstruction, the use of planar global isometric transformations is feasible; non-planar scenes, large disparity, and severe lens distortion that cannot be captured by planar models are beyond the scope of our discussion.

### 5.1. Multi-Camera Calibration

This section adopts the world coordinate system W as the unified reference frame, defines the binocular (visible light/infrared) imaging model and the relative pose between external features, and explains how to derive the global homography matrix H (VIS to IR) between images from the projection relationship from both cameras to the plane when the calibration board can be approximated as a plane.

**Figure 8:**
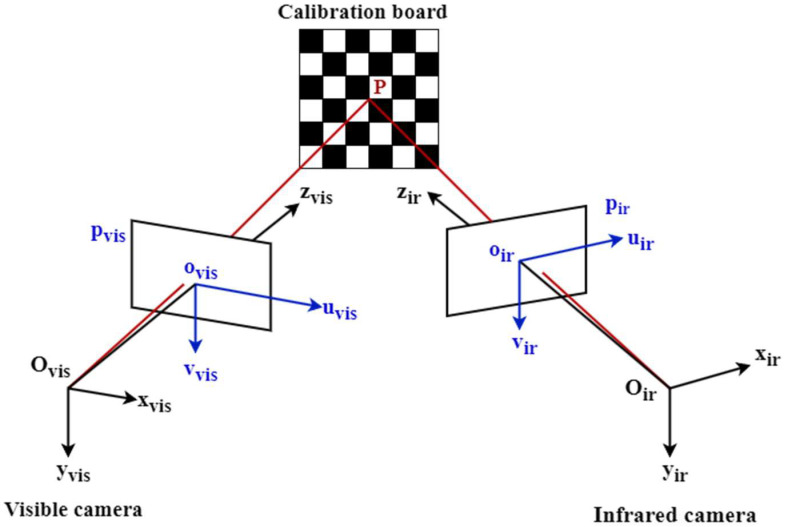
Infrared camera and visible camera coordinate model.

For any camera i (i ∈ {vis, ir}, where vis denotes a visible camera and ir denotes an infrared camera), the pinhole imaging model is adopted. As shown in Figure 8, the homogeneous forms of pixel coordinates are denoted as $p_{vis}$ and $p_{ir}$. Then, the pixel points on the visible image are represented in homogeneous coordinates as:

(1) $$p_{vis}=\left[\begin{array}{c} u_{vis}\\ v_{vis}\\ 1 \end{array}\right],$$

Equivalently, the corresponding point in the infrared image coordinates is:

(2) $$p_{ir}=\left[\begin{array}{c} u_{ir}\\ v_{ir}\\ 1 \end{array}\right],$$

The pixels in the visible image coordinate system $p_{vis}$ and the pixels in the infrared image coordinate system $p_{ir}$ satisfy the following one-to-one correspondence:

(3) $$p_{ir}\sim Hp_{vis},\;H\in R^{3\times 3},$$

where “~” denotes a difference in a nonzero scale factor in homogeneous coordinates. Denote $Hp_{vis}$ as:

(4) $$Hp_{vis}=\left[\begin{array}{c} x\\ y\\ z \end{array}\right],$$

The corresponding non-homogeneous pixel coordinates in the images captured by any camera are obtained through dehomogenization:

(5) $$u_{i}=\frac{x_{i}}{z_{i}},v_{i}=\frac{y_{i}}{z_{i}}.$$

To generate alignment results with the same resolution and field of view as the infrared image, let the infrared image size be $W_{ir}\times H_{ir}$. The aligned visible image ${\hat{I}}_{vis}$ is also defined on the same discrete grid. For each output pixel ($u_{i},v_{i}$), the sampling position in the visible image can be found via inverse mapping:

(6) $$\left[\begin{array}{c} u_{vis}\\ v_{vis}\\ 1 \end{array}\right]\sim H^{-1}\left[\begin{array}{c} u_{ir}\\ v_{ir}\\ 1 \end{array}\right].$$

If the resulting ($u_{vis},v_{vis}$) coordinate is non-integer, bilinear interpolation is applied to resample the visible-light image intensity. If the sampling position is out of bounds, zero padding or boundary copying is used (this implementation employs a unified out-of-bounds handling strategy to ensure the output size remains fixed at $W_{ir}\times H_{ir}$).

### 5.2. Results at Different Distances

**Figure 9:**
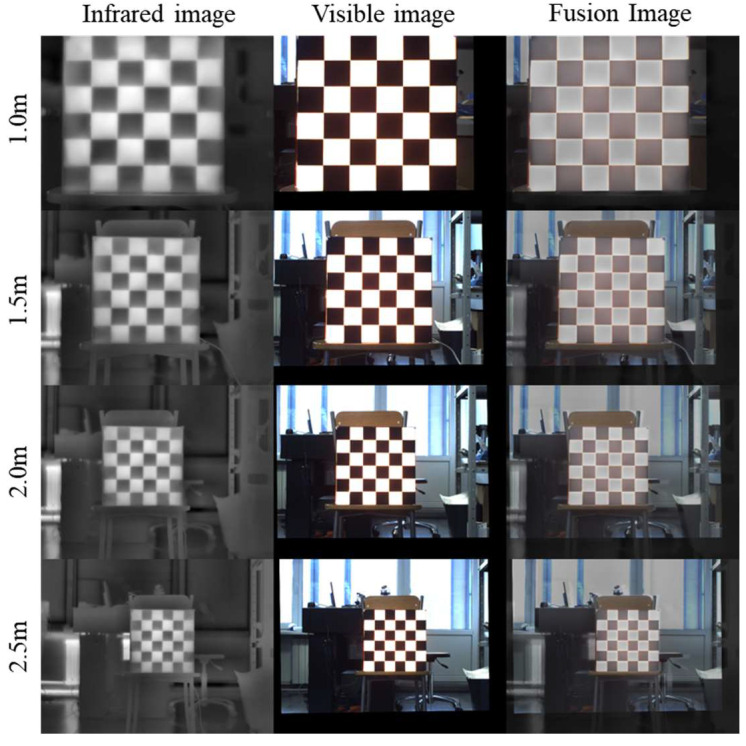
Calibration board performance at different distances.

To evaluate the stability and accuracy of the proposed calibration board calibration process at different working distances in close-range indoor environments, we set up four experimental conditions at 0.5 m increments within the 1.0 m–2.5 m distance range. At the same time, the total capture time for all images should be kept within 20 min, indoors under natural light with sufficient daytime illumination, to ensure consistent experimental conditions. At each distance, 30 consecutive pairs of IR–VIS images were captured for calibration and validation. Qualitative results are shown in Figure 9. Registration and fusion at all four distances achieved good visual consistency, with stable overlay of checkerboard edges and structures, demonstrating the method’s excellent repeatability in controlled indoor scenarios. In addition, the reprojection error evaluation was conducted under the same experimental arrangement as in Figure 5, and the resulting reprojection-error statistics remained highly consistent across the considered time intervals, which further supports the thermal stability of the proposed calibration board during active heating.

**Figure 10:**
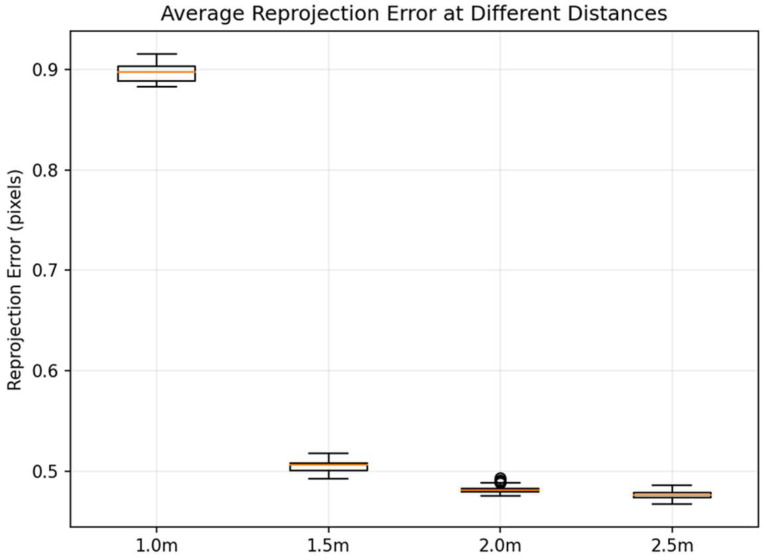
Average reprojection error at different distances. The orange line in the figure represents the median of each data set in the box plot.

For stricter quantitative evaluation, we further calculated the corner reprojection error using the same global homography matrix H across all distance conditions and plotted the distribution of average corner error per frame (Figure 10).

The results show that within the 1.5–2.5 m range, the reprojection error distribution is highly concentrated: with respective medians of 0.50 px (1.5 m), 0.48 px (2.0 m, several outliers exist, but the error remains within 0.5 pixels), and 0.47 px (2.5 m), and corresponding 95th percentiles (p95) of only 0.51 px, 0.49 px, and 0.48 px. This indicates that when the calibration board is fully within the frame and its corner points are sufficiently spaced, single-objective estimation exhibits stable geometric constraints, achieving consistent sub-pixel registration accuracy. The error long tail (characterized by p95) is notably short, demonstrating good engineering consistency.

In contrast, errors significantly increase in the 1.0 m operating condition: median = 0.89 px, p95 = 0.91 px. Additionally, only 11/30 frames were successfully utilized for global estimation at this distance (effective frame ratio ~36.7%), markedly lower than the 30/30 (100%) rate observed at other distances (see Table 3). Visual inspection of the original images reveals that the primary cause is edge clipping of the calibration board due to field-of-view limitations at a given focal length. This reduces the number of effective corner points and results in incomplete corner distribution, thereby weakening the constraints of single-response estimation and making errors more susceptible to amplification by locally unstable or outlier corner points.

The combined qualitative matching results and quantitative error statistics (median/p95) indicate that the calibration board solution proposed in this paper demonstrates overall stability under indoor close-range conditions. The 1.5–2.5 m range is the optimal recommended calibration distance interval: not only does the median remain consistently between 0.48 and 0.51 px, but the p95 also stays ≤0.52 px while achieving a 100% effective frame rate. This interval is therefore more suitable as the system’s high-precision baseline calibration setting. At 1.0 m, the calibration board is more susceptible to field-of-view cropping. To enhance stability at this distance, it is recommended to increase the calibration board size, adjust the camera’s field of view, or ensure the entire board is fully within the frame.

### 5.3. Results at Different Corners

**Figure 11:**
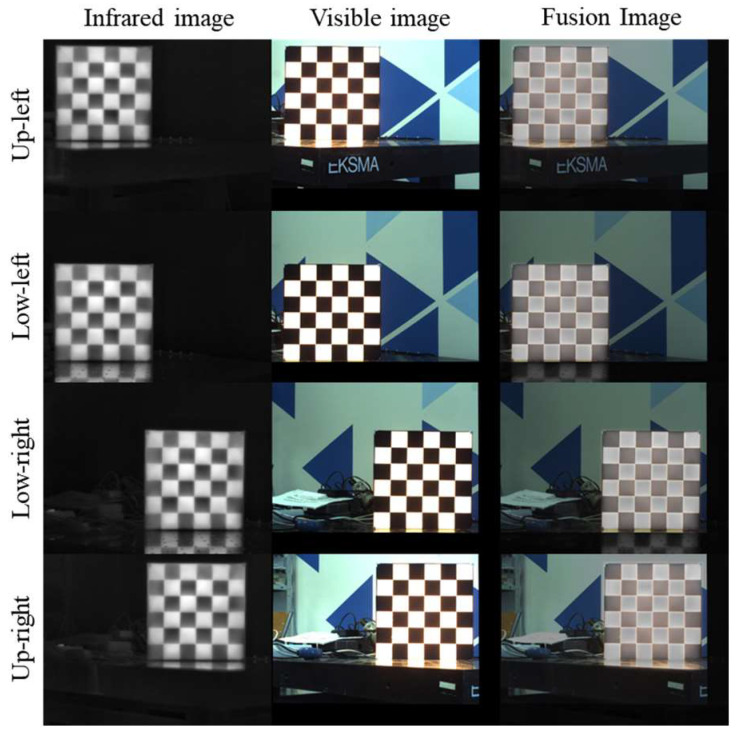
Performance of the calibration board at four corner placements.

To assess whether distortion in the lens’s peripheral regions (along with degraded imaging quality at the field of view edges) significantly impacts cross-modal registration accuracy, we positioned the calibration plate at each of the four image corners (Up-left, Up-right, Low-left, Low-right), as shown in Figure 11.

At each location, we captured 30 pairs of IR–VIS images and repeated the same corner detection and global homography estimation process. This experiment aimed to concentrate corner observations at the field-of-view edges to stress-test potential geometric errors caused by “edge distortion/dark corners/resolution degradation.”

**Figure 12:**
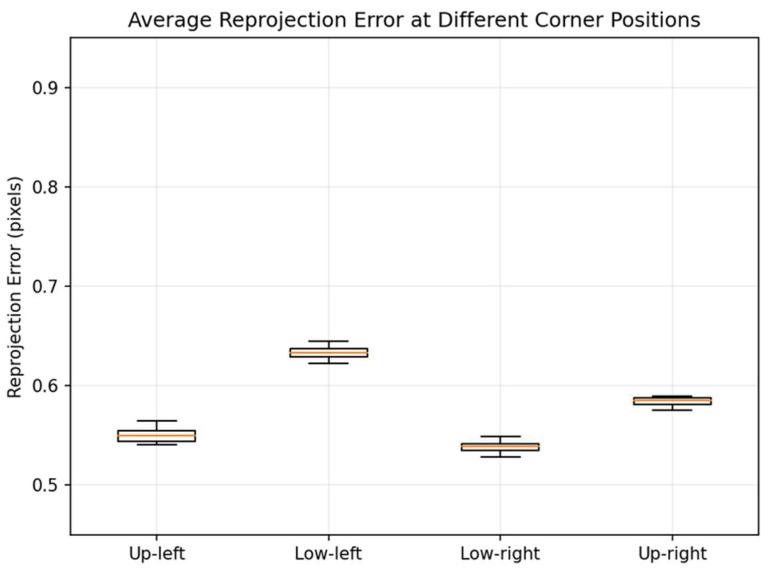
Average reprojection error at different corner positions. The orange line in the figure represents the median of each data set in the box plot.

Quantitative results are shown in Table 4. Reprojection errors at all four corner positions remained at the sub-pixel level, with relatively concentrated error distributions:

From the perspective of error statistics (Figure 12), both the median and p95 values at all four positions are less than 1 px. This indicates that under the experimental conditions of this paper, the potential distortion at the lens edges has a limited impact on the final global registration accuracy. This also demonstrates that the adopted global single-response model can effectively compensate for primary geometric mismatches (scale, translation, rotation, and minor projection distortion) under this close-range planar calibration target condition, achieving stable sub-pixel registration. Meanwhile, Table 4 shows variations in the effective frame ratio across different corner positions. Notably, at the Low-right position, only 43.3% (13/30) of frames successfully participated in estimation, suggesting that corner detection failures or instability are more likely in edge regions. This phenomenon is typically associated with degraded imaging quality at the field-of-view edges—such as reduced local contrast, minor cropping, blurring, or insufficient thermal contrast—making corner localization and consistency verification more challenging. However, on frames with sufficient detected corners, geometric estimation maintains sub-pixel accuracy.

**Figure 13:**
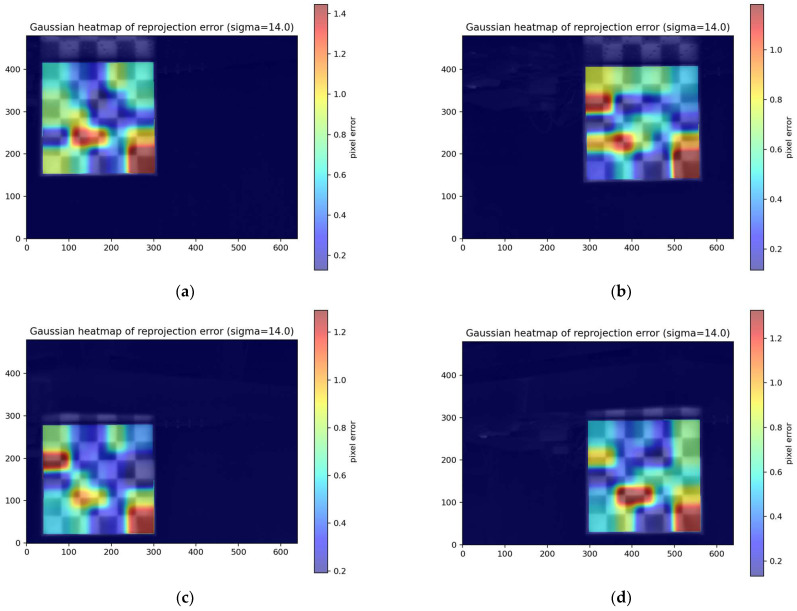
Heatmap of Reprojection Errors at Different Corners: (**a**) Up-left; (**b**) Up-right; (**c**) Low-left; (**d**) Low-right.

**Figure 14:**
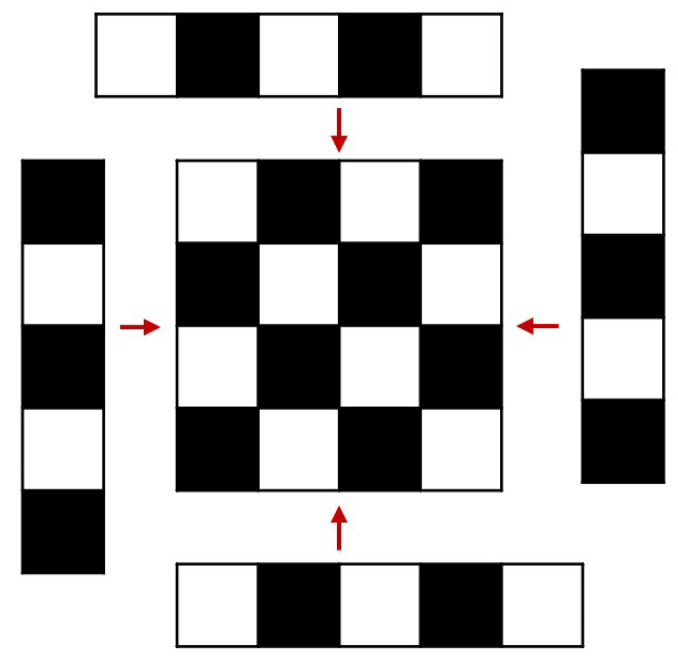
Schematic of Calibration Board Assembly. The black and white blocks represent PETG materials of different colors. The red arrows indicate the direction of the material’s assembly and the connection areas.

Additionally, we generated a heatmap of the error distribution, as shown in Figure 13. It can be observed that slightly larger errors occur at edge corners, which may also be related to the calibration plate’s assembly process: due to printer size limitations, the calibration plate was manufactured by prioritizing printing the central region (4 × 4) and then assembling it with edge modules (Figure 14). When modular assembly is required, the segments are bonded along their edges using a transparent adhesive. Under careful alignment, the in-plane seam mismatch can be controlled at the millimeter level. Nevertheless, compared with the central region, seams may introduce local geometric discontinuities and slight non-coplanarity, which can in turn lead to larger corner localization uncertainty near the periphery. In the LWIR modality, seam regions may also exhibit locally altered thermal conduction and boundary sharpness, potentially producing small corner-position biases relative to an ideal monolithic board.

These joints may introduce minute non-coplanarity, seam steps, or deviations in grid spacing, leading to systematic shifts between local corners and the ideal checkerboard pattern. Such shifts are amplified when the calibration plate is positioned near the field-of-view edge (where effective corner distribution is more localized and geometric constraints are weaker), manifesting as slightly higher edge-region errors or reduced successful frame ratios. Nevertheless, existing results demonstrate that overall error remains at the sub-pixel level, meeting the accuracy requirements for low-cost rapid calibration and fusion registration outlined in this paper.

**Figure 15:**
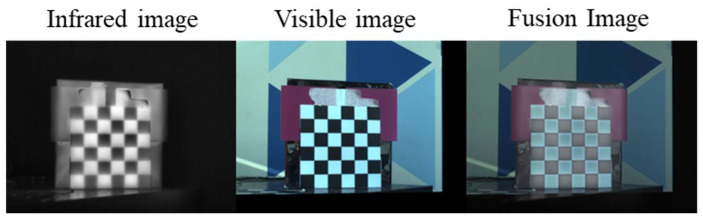
Test of the maximum single-print size calibration plate for common multi-color 3D printers (23 cm × 23 cm).

At the same time, to verify the cost-effectiveness of this experiment and investigate whether significant errors were caused by assembly, we set the calibration plate dimensions to the maximum size that a typical multi-color printer can print in a single pass (23 cm × 23 cm, Figure 15). The printing time for a single run was only 2 h and 54 min. After a simple, minute-long adjustment, we found that the re-projection error of the calibration plate could be further reduced to 0.36 pixels—a value already very close to that achieved by other high-precision methods [13]. At the same time, our production costs and assembly time are lower, validating the cost-effectiveness of our method in terms of both assembly and manufacturing. Compared to the previous modular assembly version, the precision of the monolithic print is significantly higher; therefore, monolithic printing is the preferred option. However, when the laboratory requires calibration board dimensions that exceed the printer’s size limitations, the assembly method shown in Figure 14 is recommended.

To support the cost-effective and rapid-deployment nature of the proposed calibration target, we report manufacturing requirements in terms of measurable and reproducible components rather than region-dependent prices. For a representative single-piece board (approximately 23 × 23 cm, 6 × 6 grid), the total filament usage is about 86.23 g (27.78 g of white PETG material and the remainder black PETG material), and the printing time is approximately 2 h 36 min under our fabrication settings. This workflow relies only on consumer-grade additive manufacturing and avoids specialized machining processes typical of ceramic infrared targets (e.g., precision drilling or custom fabrication), thereby reducing fabrication overhead and material waste while enabling fast iteration. In addition, the thermal contrast required for LWIR observability is achieved using a simple 60 W incandescent lamp, which is inexpensive and readily available compared with controlled, homogenized heating systems. Overall, the proposed approach provides an accessible and reproducible target-fabrication route suitable for engineering calibration tasks where rapid turnaround and low equipment dependence are priorities.

The above experiments demonstrate that the proposed chessboard-based workflow can achieve sub-pixel reprojection error in close-range indoor conditions, even when the target is placed near the image boundaries. Through verification, it has been found that this method can satisfy the calibration requirements for infrared and visible camera systems. At distances between 1.5 and 2.5 m, calibration errors remain within sub-pixel accuracy. However, compared to other complex methods [19,21,22], there is still room for improvement in accuracy. Nevertheless, the primary advantage of this method lies in its ability to rapidly design and manufacture based on actual camera system parameters, enabling rapid calibration. For scenarios requiring quick verification of camera systems or controlling experimental costs, this method is undoubtedly the optimal choice for calibrating infrared-visible camera systems.

### 5.4. Camera Calibration Parameters

Using the infrared and visible-light cameras mentioned earlier, 30 sets of calibration images taken from different positions were selected. The calculated intrinsic parameters, distortion coefficients, and global homography matrix are listed in Table 5 and Table 6 and Equation (7), respectively.

Regarding the results of the plane registration, the global homography matrix $H_{vis\to ir}$ can be calculated as follows:

(7) $$H_{vis\to ir}=\left[\begin{array}{ccc} 0.4698877 & 0.0083814 & -19.1571\\ -0.0003905 & 0.4686138 & -13.5086\\ 1.5822*10^{-5} & 1.4632*10^{-5} & 1 \end{array}\right],$$

## 6. Conclusions

This study developed a rapid deployable and reproducible calibration board process for practical geometric registration of infrared and visible-light sensors. This calibration board maintains a stable state under prolonged exposure to high temperatures, and the black and white squares remain clearly visible under an infrared camera, with an average temperature difference of 8.52 °C. In a controllable indoor environment, the proposed dual-modal checkerboard and corner point estimation based on the homography matrix achieved stable sub-pixel alignment at a working distance of 1.5–2.5 m, with a median re-projection error of 0.47–0.50 px, and the 95th percentile was lower than 0.51 px, with an effective frame rate of 100%. However, at 1.0 m, due to field-of-view truncation, corner point detection was insufficient, and the median error increased to 0.89 px, indicating that ensuring the complete visibility of the target is the primary condition for maintaining a stable calibration.

The spatial error assessment showed that all four corner point layouts maintained sub-pixel accuracy (median 0.54–0.63 px), and edge distortion was not the main error source. The proposed calibration board and process, through structured parametric rapid modeling and design-oriented manufacturing, reduced the setup threshold for the IR-VIS system. For future work, the following three directions present significant potential for in-depth exploration: First, designing optimal calibration board array configurations based on 3D printer size and camera parameters to eliminate assembly induced errors; second, employing different engineered material combinations to enhance calibration image contrast. Finally, this calibration board can be configured not only as a Zhang’s Chessboard pattern but also switched to alternative calibration patterns as needed to accommodate diverse calibration scenarios. The current evidence supports the practical effectiveness of the proposed design for calibration in controlled near-range deployment, while systematic thermal robustness characterization (e.g., CNR/gradient statistics, repeatability across heating cycles, and lamp/airflow sensitivity) is left unaddressed and will be considered in follow-up work or supplementary evaluations.

## Figures and Tables

**Table 1:** Infrared and visible multispectral camera module and associated optical parameters.

Information	Infrared Camera	Visible Camera
Model	FLIR—A615	Basler aca1300-30gc
Wavelength range	7.5–14 μm	450–620 nm
Focal length	24.6 mm	12 mm
Pixel size	17 µm	3.75 µm
Resolution	640 × 480	1294 × 964

**Table 2:** Calibration board production parameters.

Information	Black Square	White Square
Thickness	1.2 mm	0.8 mm
Material	SUNLU PETG	
Square size	50 × 50 mm	
Layer height	0.1 mm	
First layer height	0.1 mm	
Interlayer color	White	
Interlayer range	0.6–0.8 mm	

**Table 3:** Qualitative Matching Results and Quantitative Error Statistics at Different Distances (Median/ 95th percentile).

Distance	Frames Used/Total	Median (px)	p95 (px)
1.0 m	11/30	0.89	0.91
1.5 m	30/30	0.50	0.51
2.0 m	30/30	0.48	0.49
2.5 m	30/30	0.47	0.48

**Table 4:** Qualitative Matching Results and Quantitative Error Statistics at Four Corner Positions (Median/95th percentile).

Position	Frames Used/Total	Median (px)	p95 (px)
Up-left	30/30	0.55	0.55
Low-left	26/30	0.63	0.64
Low-right	13/30	0.53	0.54
Up-right	30/30	0.58	0.58

**Table 5:** The intrinsic matrix of the LWIR camera and VIS camera (pixel).

Camera	$f_{x}$	$f_{y}$	$u_{0}$	$v_{0}$
LWIR (IR)	1568.20	1567.94	322.59	233.45
VIS	3238.08	3238.11	540.44	346.44

**Table 6:** The distortion coefficients of the LWIR camera and VIS camera.

**Camera**	$k_{1}$	$k_{2}$	$k_{3}$	$p_{1}$	$p_{2}$
LWIR (IR)	0.3882	0.1745	0.0010	−0.00194	0.00251
VIS	−0.5103	0.1317	0.0143	−0.00427	−0.00458

## Data Availability

The data presented in this study are openly available in MakerWorld parametric model editing website: https://makerworld.com/zh/makerlab/parametricModelMaker?pageType=home&from=makerlab (accessed on 11 April 2026). Calibration board design and automatically generated code: https://github.com/Rango-shao/Calibration_board_generation.git (accessed on 11 April 2026). Infrared and visible camera picture acquisition and calibration code: https://github.com/Rango-shao/Infrared-visible-camera-calibration.git (accessed on 11 April 2026).
